# Significant Liver Atrophy Due to Vascular Compromise Associated With Adult Congenital Diaphragmatic Hernia

**DOI:** 10.7759/cureus.17158

**Published:** 2021-08-13

**Authors:** Nitin Agarwal, Manoj K Dokania, Gyan R Kumar, Dharmateja G Manda, Anil K Singh Rana

**Affiliations:** 1 Transplant Unit, Department of Surgery, Atal Bihari Vajpayee Institute of Medical Sciences (ABVIMS) and Dr. Ram Manohar Lohia Hospital (RMLH), Delhi, IND

**Keywords:** adult congenital diaphragmatic hernia, liver atrophy, vascular compromise, outflow obstruction, multi-visceral content

## Abstract

Diaphragmatic hernia in adults is mostly post-traumatic in origin, and rarely congenital. In both situations, the right side is less commonly involved due to the protection offered by the liver and earlier closure of the right pleuroperitoneal canal. A congenital diaphragmatic hernia may present in adulthood with multi-visceral contents, of which the liver is an extremely rare content, mentioned only in a few previous reports. A herniated liver may mimic a pulmonary tumor and may be completely atrophic due to sustained compression of the venous outflow. Careful operative planning is essential to identify and reduce the liver, along with other contents. We are reporting two adults with a congenital diaphragmatic hernia, with multi-visceral contents and an atrophied liver. The first patient was a 28-year-old man with a remote history of trauma found to have a large right diaphragmatic hernia on imaging. The right liver was completely atrophied due to right hepatic venous compression, while the left liver underwent massive hypertrophy and rotation of the left portal axis. Exploratory laparotomy and reduction of contents, along with mesh repair, were accomplished with satisfactory results. The second patient was a 26-year-old man with Down’s syndrome detected to have multiple bowel loops in the right thorax on imaging. At laparoscopy, a Larrey’s type of Morgagni hernia with a right paramedian defect was found. The left liver was atrophied into a leaf-like appendage due to possible portal obliteration and was dissected away from the diaphragm edge. Appropriate mesh repair was completed by a minimally invasive technique.

## Introduction

Congenital diaphragmatic hernia (CDH) occurs in less than 1 in 25,000 births, and presentation in adulthood is seen in 5%-25% of cases [1]. Diaphragmatic hernia in adults may also be post-traumatic; in both of the above situations, left-sided preponderance (70%-90%) is noticeable. This is attributed to the protection offered by the liver, and by the earlier closure of the right pleuroperitoneal canal [2,3]. CDH in the neonatal period often causes cardiorespiratory distress and mortality, while in adults it is mostly insidious and rarely acute in presentation [3,4].

Congenital diaphragmatic hernia often leads to multi-organ herniation of contents into the thorax, hypothesized to be due to hypoplasia and malposition of the hemidiaphragm, facilitated by altered neural crest migration and organ hypoplasia [1,5]. In adult CDH, the liver is a rare content, mentioned in only a few case reports [2,3,5-8]. It is even rarer to find a significant portion of the liver rendered atrophic due to vascular compromise [3,5,9]. We report two cases of adults with diaphragmatic herniae and liver atrophy due to vascular compromise.

## Case presentation

Case 1

**Figure 1:**
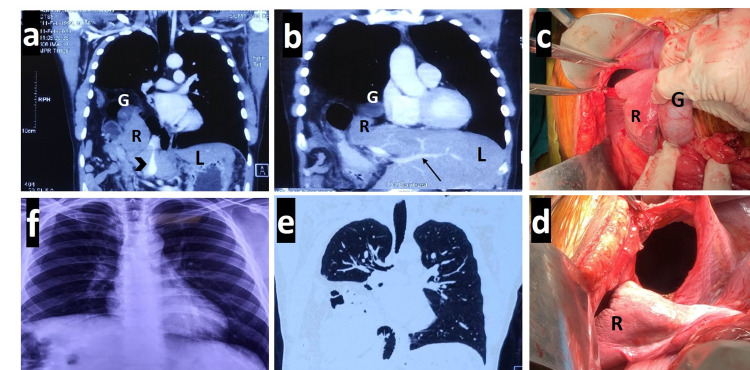
Clockwise from upper left: (a) Coronal contrast CT section of patient 1 showing multiple abdominal viscera entering the right thorax, namely, the colon and small bowel, gallbladder (marked G), and atrophied right liver (marked R). The hypertrophied left liver (marked L) and the middle hepatic vein (marked with black arrowhead) are shown. The right hepatic vein is probably obliterated. (b) Another coronal CT image depicting the complete extent of left liver hypertrophy with the rotated left portal vein axis (marked with black arrow). (c) and (d) Operative images showing the well-defined right posterolateral defect after reduction of contents, with the atrophied right liver (R) and the gallbladder (G). (e) Pre-operative plain CT with multiple viscera in the right hemithorax. (f) Postoperative chest radiograph with complete lung expansion on day seven. CT - computed tomography.

A 28-year-old man presented to the surgery clinic with a six-month history of right-sided chest pain and fullness after meals. The patient reported a motor accident three years back with injury to the right chest, which resolved with expectant management after five days. Present examination revealed decreased air entry and audible bowel sounds in the right chest, suggestive of a diaphragmatic hernia. Chest radiographs suggested the presence of a raised right hemidiaphragm and gas-filled bowel loops in the right hemithorax. All hematological and biochemical blood tests, including liver function tests and coagulation profile, were normal. CT thorax revealed a large posterior right diaphragmatic defect of 6 x 6 cm, with herniation of many intra-abdominal contents into the right thoracic cavity. These were an atrophic right liver (segments 5-8) with the gallbladder, a part of the distal stomach and pancreatic head, the entire right colon with the appendix, and the small bowel (Figures 1a, 1b). The left liver was greatly hypertrophied and recognizable only by the presence of the rotated left portal vein in the umbilical fissure and the presence of the gallbladder in the thorax. Anticipating a conversion to thoracotomy due to a kinked right liver outflow, the patient was planned for open surgery and not laparoscopy. A midline laparotomy was performed and the diaphragmatic surface was exposed using the Bookwalter^TM^ retractor system. An irregular, 6 x 6 cm defect was identified in the posterior right diaphragm with no sac. All the contents were gently reduced, taking special care to reduce the atrophied right liver and gallbladder twisted upwards with the right hepatic vein. Almost 6 feet of the small bowel, the entire right and transverse colon, parts of the distal stomach-duodenum, and pancreatic head were reduced back into the abdominal cavity (Figures 1c, 1d). Lung expansion was normal. After clearing 5 cm around the defect, it was closed with interrupted polypropylene sutures and a 15 x 15 cm polypropylene-oxidized cellulose composite mesh was placed over it. The abdomen was closed after the insertion of an intercostal drain. The right lung expanded satisfactorily in the postoperative period (Figure 1f). The patient was discharged after a week and did well six months later.

Case 2

**Figure 2:**
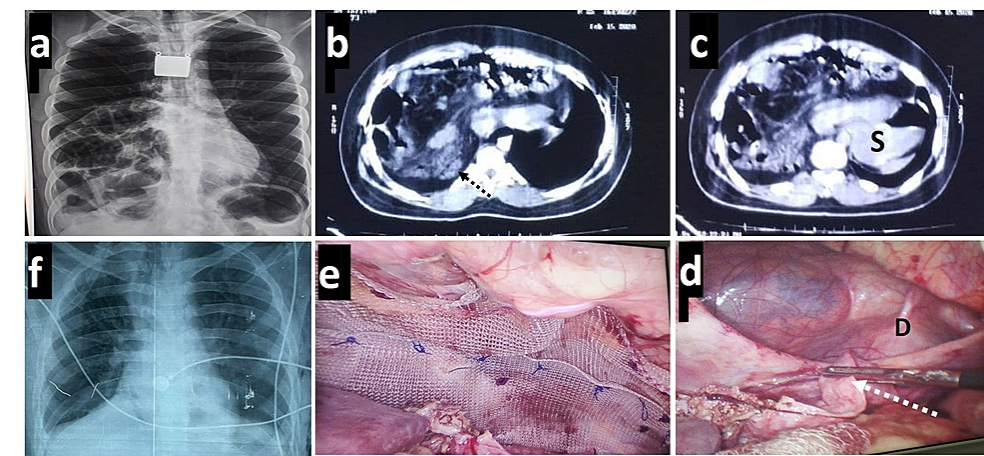
Clockwise from upper left: (a) Pre-operative chest radiograph with multiple viscera in the right hemithorax. (b) Cross-sectional contrast CT suggestive of multiple viscera in the right hemothorax and a linear hyperdensity (marked with dashed black arrow) possibly representing the atrophied left liver. (c) Similar CT image showing the stomach (marked S) entering the thorax through the defect and falling to the left. (d) Operative image with the large defect (D) with indistinct ventral edges. The dorsal edge is being cleared by dissecting away the atrophied left liver (marked with dashed white arrow). (e) Defect closure and a large overlapping mesh has been placed. (f) Postoperative chest radiograph with complete lung expansion on day seven. CT - computed tomography.

A 26-year-old man diagnosed with Down’s syndrome since birth presented to the surgery clinic with a three-month history of abdominal pain, nausea, and post-prandial vomiting. The patient had bilateral strabismus, moderate mental retardation, and left undescended testis since birth. Bowel sounds were audible in the right lower thorax; however, no abdominal lump was palpable. Chest radiographs suggested adequate expansion of both lungs with a high diaphragm and dilated bowel loops in the right paramedian thorax. Contrast-enhanced CT revealed normal lung fields with a possible defect in the right anterior paramedian diaphragm; the entire right colon along with omentum and part of fundus of stomach was seen to lie in the right lower paramedian thorax (Figures 2a-2c). The patient was taken for laparoscopic repair with three ports at the infra-umbilical and right and left mid-clavicular locations. A large oval defect of 6 x 5 cm was found in the anterior right diaphragm close to the midline involving the falciform ligament. The ventral rim of the diaphragm was poorly developed. A well-formed hernial sac was seen with the following contents: the right colon with the appendix, a part of the gastric fundus and omentum, about 3.5 feet of the small bowel, and the left lateral liver section twisted on its horizontal axis. The liver was a surprise finding not reported on CT earlier. Due to the liver section rotation, segment 3 was atrophied into a leaf-like appendage while segment 2 was partially hypertrophied and elongated (Figure 2d). The right paramedian location of the defect and contents had pushed the pericardium slightly more to the left (Larrey’s variety of Morgagni hernia). The contents were carefully reduced with atraumatic laparoscopic graspers; the liver section was reduced, de-rotated, and dissected away from the defect margins up to 5 cm. The defect was closed with absorbable barbed sutures using the anterior abdominal fascia and the posterior defect lip, incorporating the sac eliminating the dead space. A 15 x 15 cm lightweight polypropylene-polyglactin combination mesh was fixed over the defect area with absorbable tacks and sutures. The patient recovered well and did not experience any complications till recently, i.e., two months of follow-up. 

## Discussion

In adults, post-traumatic diaphragmatic herniation is more common than CDH. However, the history of trauma may be remote or incidental, and many patients may be erroneously diagnosed as CDH. A few authors have discussed the occurrence of diaphragmatic liver herniation of varying degrees, usually small, following remote presumed trauma [4,10-12]. These were definitely not congenital herniae, and the remote trauma was attributed to catamenial pneumothorax or endometriosis since most of these patients were middle-aged females [4,12]. The primary suspicion on imaging was a suspicious pulmonary nodule, which warranted resection in two of these cases [10,12]. 

In our first case, we initially felt that it was a post-traumatic diaphragmatic hernia, due to an absent hernial sac and absence of pulmonary hypoplasia. However, the multi-visceral contents and the atrophied liver were more suggestive of congenital posterolateral (Bochdalek) hernia. The sac is known to be absent in some cases of Bochdalek hernia [6,7]. The two issues unique to both our patients with CDH, in view of the liver atrophy, were identifying the cause and the decision for resection. Authors have reported different cases based on the extent of liver involvement. Peker et al. reported a case of a 75-year-old man, presenting six years after trauma with three sections of the liver herniated (segments 4-8). However, there was no liver atrophy, and the contents were carefully reduced [13]. Dharmik and colleagues reported an 18-month-old boy with CDH and left lateral liver section aplasia. They suggested that vascular or pressure effects could have occurred during the intrauterine period. We also believe a similar event to have occurred in our adult patients [9]. Gurrado et al. observed CDH with multiple organ defects including left hepatic hypoplasia in a 64-year-old woman. They suggested that delayed diaphragmatic development contributed to scanty epithelial cells or hepatoblasts, preventing proper liver growth [5]. Zenda and colleagues found complete atrophy of the middle liver section (segment 4) in a 69-year-old man with Bochdalek hernia. They presumed it to be due to vascular compression and suggested that this atrophy further facilitated the migration of other contents upwards [3]. Banchini and colleagues reported a right Bochdalek hernia with rotation of the entire right liver into the thorax with occlusion of the right and middle hepatic veins. A natural right to left shunt anterior to the umbilical recess prevented atrophy of the herniated right liver. The authors suggest pre-operative careful scrutiny for such a shunt to avoid inadvertent vascular injury [6]. All these accounts suggest that a vascular compromise of the outflow tract over a prolonged period leads to atrophy of the liver in CDH. Efforts should be made to identify vascular kinks, collateral vessels, and atrophy-hypertrophy of the liver segments on imaging. These signs would indicate vascular compromise. Ayane et al also found three accessory liver lobes, probably representative of the right liver, in an adult CDH. While they performed the appropriate resection of the incarcerated bowel, no intervention was performed for the liver. They believed that it was not necessary due to substantial hypertrophy of the remnant [7]. We have followed the same approach, i.e., the atrophy-hypertrophy was not attributable to biliary ‘maldrainage’ as seen in cholangiocarcinoma or strictures, and could be left without intervention. Resection is required only when the liver is completely unviable, or there is suspicion of malignant change (vide supra). In our second case, prior imaging did not identify the small left liver, and it was seen after laparoscopy. Due to its small size and leafy morphology, it could be separated without event.

## Conclusions

In conclusion, if a substantial portion of the liver is seen as a content of an adult CDH, it is important to identify the vascular anatomy of the liver. Efforts should be made to identify vascular kinks, collateral vessels, and atrophy-hypertrophy of the liver segments on imaging. These signs would indicate vascular compromise. Most such cases would need an open approach due to the potential need for careful dissection of the retrocaval hepatic venous confluence. Selected cases can be managed laparoscopically.
